# The Different Faces of Social Tolerance: Conceptualizing and Measuring Respect and Coexistence Tolerance

**DOI:** 10.1007/s11205-021-02724-5

**Published:** 2021-06-16

**Authors:** Evi Velthuis, Maykel Verkuyten, Anouk Smeekes

**Affiliations:** Department of Interdisciplinary Social Science, European Research Centre On Migration and Ethnic Relations, Utrecht University, Padualaan 14, 3584 CH Utrecht, The Netherlands

**Keywords:** Social tolerance, Respect, Coexistence, Prejudice, Minority rights, Immigrants

## Abstract

In the theoretical literature on tolerance a distinction is proposed between coexistence and respect tolerance. In three studies with four national samples of Dutch majority members, we demonstrate that these two forms of tolerance can be distinguished empirically in relation to different immigrant target groups. The findings of all studies further show that the more principled respect tolerance was negatively associated with prejudice towards immigrants, and positively associated with the acceptance of concrete minority practices, above and beyond prejudice. However, the positive association between respect tolerance and acceptance of practices was weaker for people who were more strongly concerned about the continuity of their national cultural identity. Overall, the more pragmatic coexistence tolerance was found to have no independent association with prejudicial feelings and with the acceptance of minority practices. The findings indicate that stimulating respect tolerance might be particularly helpful for improving intergroup relations in culturally diverse societies.

**Supplementary Information:**

The online version contains supplementary material available at 10.1007/s11205-021-02724-5.

## Introduction

Tolerance is increasingly promoted in national, international, and organizational settings for establishing multicultural justice and peaceful coexistence. Leaders from various countries, the European Union, the United Nations (UN), and non-governmental organizations have all emphasized the importance of policies that promote tolerance which, among others, has resulted in the UN *International Day for Tolerance* and a ‘*European model law for the promotion of tolerance and the suppression of intolerance*’ (European Council on Tolerance & Reconciliation, 2015). Similarly, religious and civic associations as well as schools worldwide promulgate social tolerance^1^ as a critical aspect of social life, often as a response to increasing diversity resulting from continuing immigration. In western societies, tolerance is frequently discussed in relation to the accommodation of immigrant groups and Muslim minorities in particular (Verkuyten et al., 2019).

Tolerance is a counteracting force against suppression and negative interference, allowing dissenting others the right to lead the life that they want (Norris, 2002). It is a particular type of liberty that requires the application of a notion of freedom to those who are disliked or to practices and beliefs one disapproves of (Cohen, 2004; King, 2012). However, people can have different reasons for being tolerant: for *why* they accept that dissenting others can affirm their views and live their life accordingly. Yet, little empirical research has explicitly considered these reasons to tolerate (see Hjerm et al., 2019; Klein & Zick, 2013). Two main reasons are, first, avoiding societal conflicts and, second, respecting the equal standing and rights of others (Verkuyten et al., 2019). These two reasons are similar to two forms of tolerance that have been put forward in a historical and conceptual analysis by Forst (2013) and which can be present in society simultaneously, namely coexistence tolerance and respect tolerance^2^ (Forst, 2017). These forms of tolerance differ in how strongly they are based on pragmatic rather than principled concerns – corresponding to principled and pragmatic ways of reasoning about societal issues (e.g., Colombo, 2021) – and therefore might have different implications for intergroup relations in culturally diverse societies.

The aim of the current research is to advance the study of social tolerance by examining these two forms of tolerance among four national samples of majority group members in the Netherlands. We examined the meaning and distinctiveness of both forms of tolerance in relation to dissenting others in general, towards different types of minority and immigrant target groups, in relation to concrete practices of Muslims (Study 3), and in relation to prejudice (Studies 1–3). As tolerance entails accepting the freedoms of others that one dislikes or objects to, considering the different forms of tolerance and their associations with prejudice provides insight into the issue to what extent tolerance and prejudice co-occur (Fairlamb & Cinnirella, 2020; Gibson, 2006). Additionally, also majority members who are more tolerant of minorities and immigrants might want to maintain a sense of in-group continuity (Smeekes & Verkuyten, 2015). Therefore, in Study 3, we also examined whether the expected positive tolerance-acceptance association is weaker when majority members are more concerned about the continuation of their in-group culture and identity.

### Forms of social tolerance

Tolerance implies the notion that dissenting others, and minorities in particular, should be able to live the life that they want (Norris, 2002). In general, people might have more principled or more pragmatic reasons for putting up with what they dislike or object to (Verkuyten et al., 2019).

*Respect tolerance* is based on the principled belief that all citizens are autonomous individuals who have equal rights. Although there are sometimes ‘deep’ cultural differences in ways of life, minority members are tolerated because they are respected as equal, autonomous citizens with the same dignity, rights and civil liberties (Hjerm et al., 2019; Simon, 2007).

*Coexistence tolerance* implies a more pragmatic acceptance of minority groups’ ways of life in order to avoid conflicts and to find and maintain peaceful cohabitation (Kirchner et al., 2011). Here the focus is not so much on the rights of minority groups but rather on living together. Coexistence tolerance is considered instrumental to the attainment of the value of maintaining social harmony and peace, and things that go against this should not be tolerated.

Going beyond initial empirical research in Germany (Klein & Zick, 2013), we examined these two conceptualizations of tolerance in relation to various minority target groups, and empirically tested the prediction that these two forms of tolerance are distinct understandings among the public (*Hypothesis 1a*). Furthermore, using an experimental design, we examined whether both forms have a similar distinctive meaning in relation to different immigrant groups. The two forms of tolerance represent general reasons for why people tolerate ‘others’ and do not refer to specific immigrant groups or specific practices and lifestyles. This should make comparisons across groups possible (Hjerm et al., 2019). If people think that minority members should be allowed to live the life that they want for pragmatic or more principled reasons, they are likely to distinguish these two reasons consistently across groups. This means that we expected that the two forms of tolerance can be empirically distinguished in relation to different types of immigrant groups, with each form having a similar meaning in relation to these groups (*Hypothesis 1b*).

Distinguishing the two forms of tolerance in relation to different immigrant target groups does not have to mean, however, that the level of endorsement is similar across the groups. For example, the endorsement of coexistence tolerance might be stronger in relation to immigrant groups that are perceived to be culturally more different or as posing a greater challenge to societal cohesion than other immigrant groups. This could mean, for example, that Dutch majority members emphasize coexistence tolerance more strongly in relation to Muslim immigrants and non-Western immigrants, as compared to non-Muslim and Western immigrants. Yet it is also possible that the different reasons for being tolerant are not immigrant group-specific. Some research suggests that anti-immigrant attitudes are quite similar towards different groups of migrants (e.g., Kinder & Kam, 2009; Sniderman et al., 2004), because these attitudes would be driven by underlying psychological predispositions and ideological beliefs. The same might be true for the two forms of tolerance which both emphasize, although for different reasons, the general importance of minorities being able to live the life that they want. Therefore, we explored whether the level of endorsement of the two forms of tolerance depends on the particular type of immigrant target group.

### Tolerance and Prejudice

Tolerance and prejudice are theoretically and empirically distinct phenomena (e.g., Gibson, 2006; Hjerm et al., 2019; Klein & Zick, 2013; Verkuyten et al., 2020). People can have negative beliefs and feelings about a group but nevertheless support the civil liberties of that group to live the life that they want. They are capable of accepting practices and beliefs of those whom they dislike, disapprove of, or disagree with. Furthermore, people can reject specific practices (e.g., ritual slaughter of animals) of a group (e.g., Jews, Muslims) to whom they have neutral or even positive feelings (Hurwitz & Mondak, 2002; Van der Noll, 2014). Consequently, previous research has found mixed results for the relation between tolerance and prejudice (e.g., Fairlamb & Cinnirella, 2020). Since the two forms of tolerance differ in their general reasons for allowing minority groups to live their own way of life, the association between tolerance and prejudice might depend on the specific form of tolerance. This would further validate the meaningfulness of making a distinction between the two forms, and shed light on the extent to which tolerance and prejudice co-occur.

Respect-based tolerance focuses on the civic status of minority members as autonomous members of society. When people respect members of another group as equals, it is likely that they are not strongly negative towards this group. In research among Tea Party supporters (Simon et al., 2018), it was found that respect for homosexuals and Muslims as equal fellow citizens goes together with more positive attitudes towards those out-groups. Furthermore, a study in Sweden found respect-based tolerance to be associated with lower prejudice towards immigrants (Hjerm et al., 2019; but see Klein & Zick, 2013). Therefore, we expected that stronger endorsement of respect tolerance is associated with lower prejudice towards (immigrant) minorities (*Hypothesis 2*).

The coexistence conception of tolerance focuses on societal harmony and the peaceful functioning of society. Its instrumental and more conditional nature makes it morally less imperative than respect tolerance. Coexistence tolerance is a question of societal risks and opportunities in a given time and place, and emphasizes that majority and minority groups live together in society. This might imply a less clear and robust association with prejudice towards minorities. For instance, Klein and Zick (2013) found no independent relation between coexistence tolerance and prejudice. In some situations also people with prejudicial feelings might be willing to accept others in order to prevent conflicts and secure peaceful coexistence. They may think that in given circumstances it is in society’s best interest to tolerate minorities to live the life that they want. However, in other situations prejudiced people might feel that suppression and exclusion rather than tolerance is in the best interest of society. Thus, we will explore how coexistence tolerance relates to prejudicial feelings, and whether it is associated with prejudice independently of respect tolerance.

Additionally, we examined whether the relations between the two forms of tolerance and prejudice are similar for four types of immigrant target groups (Western, non-Western, Muslim, non-Muslim). It is possible, for example, that the coexistence-prejudice association is more pronounced for immigrant groups that are considered culturally more dissimilar, than for other groups for instance accepted for pragmatic reasons. However, for principally-based respect tolerance, the relations with prejudice are likely to be the same across different types of immigrant groups.

### Tolerance and the Acceptance of Minority Practices

Research has shown that there often is a difference in the way in which people judge abstract reasons and general notions in comparison to concrete cases and specific situations (Dixon et al., 2017). It is one thing to agree with the general notion that minority members have the freedom to live the life that they want, but another to accept, for example, the ritual slaughter of animals or Muslim teachers in public schools wearing a headscarf. It is around concrete issues (e.g., dress code, religious education, language use, dietary requirements, mosque building, parenting styles) that ways of life collide and the need for acceptance of cultural diversity arises. Therefore, it is important to examine whether the two forms of tolerance are associated with the acceptance of concrete minority practices, above and beyond group-based prejudice. Study 3 focuses on tolerance of Muslim minority practices,^3^ as the immigrant-origin group that is most strongly and most negatively debated in Dutch society (Andriessen, 2016). Demonstrating that the two forms of tolerance are independently related to the acceptance of these practices would provide further support for the meaningfulness of the distinction between these conceptualizations of tolerance.

With regard to respect tolerance, people may think that some controversial minority practices (e.g., Muslim public school teachers wearing a headscarf) are without merit, but still accept others to practice these because they respect them as autonomous members of society with equal rights. Simon and Schaefer (2018) found that accepting dissenting practices and beliefs is likely when there is respect for others as fellow, equal citizens (see also, Simon et al., 2018). Thus, respect tolerance can be expected to be associated positively with the acceptance of Muslim minority practices, above and beyond prejudicial feelings (*Hypothesis 3a*).

Coexistence tolerance focuses on the values of peace and societal harmony, which might be a reason for allowing minority members to live the life that they want (Haidt, 2012). A pragmatic tolerant person may prefer to refrain from negatively interfering with dissenting minority practices because they think that an intolerant reaction might cause social tensions, resistance and conflicts. Thus, coexistence tolerance also can be expected to be associated positively to the acceptance of concrete Muslim minority practices, on top of prejudice (*Hypothesis 3b*).

### The Role of Identity Continuity Concern

Tolerance differs from indifference (‘who cares’) and relativism (‘anything goes’) since there are boundaries to what can and should be accepted (Cohen, 2004; Fairlamb & Cinnirella, 2020; King, 2012). Perceived in-group continuity has been discussed as an important boundary condition of what is acceptable (Verkuyten et al., 2019). Things that threaten or undermine the continuity of the in-group culture and identity are difficult to accept. This means that the expected positive associations between the two forms of tolerance and the acceptance of minority practices might depend on concerns about in-group identity continuity.

Research indicates that people want to maintain a sense of in-group continuity and more strongly strive for or are more concerned for maintaining it when such a sense is challenged, for instance by societal changes due to immigration and increasing cultural diversity (Smeekes & Verkuyten, 2015; Vignoles, 2011). Some immigrant cultural practices and expressive rights can be perceived as undermining the continuity of the national cultural identity, also for majority members who endorse the general notions of respect and coexistence tolerance. Experimental research has shown that people reject minority practices which are considered to contradict society’s normative and moral ways of life (e.g., Helbling & Traunmüller, 2018; Sleijpen et al., 2020). Thus, beliefs about tolerance might not always translate into acceptance of concrete practices if people are concerned about the continuity of their in-group’s identity. All in all, we expected that the association between the endorsement of (a) respect tolerance and (b) coexistence tolerance with the acceptance of Muslim minority practices is less strong for majority members who are more concerned about the continuity of their in-group’s cultural identity (*Hypothesis 4a and 4b*).

### Overview

We investigated our predictions in three studies using four datasets collected among national samples of Dutch majority members. *Hypotheses 1a* and *2* were tested with all four samples in order to examine whether the expected empirical distinction between the two forms of tolerance exists. Additionally, we investigated whether the expected differential associations with prejudice replicates across samples, and for prejudicial feelings towards dissenting others in general (Study 1a), towards cultural minorities (Study 1b), towards different immigrant groups (Study 2), and towards Muslim immigrants (Study 3).

In Study 2 we used an experimental design to test whether the meaning and endorsement of the two forms of tolerance is immigrant-group specific or rather similar across immigrant groups (*Hypothesis 1b).* Specifically, we varied the immigrant category about which participants had to answer the tolerance questions across the conditions, comparing Western versus non-Western immigrants, and Muslim versus non-Muslim immigrants. Also, we explored whether the tolerance-prejudice relations are similar across these four target groups. Lastly, in Study 3 we examined whether the two forms of tolerance are positively associated with the acceptance of concrete Muslim minority practices (*Hypothesis 3a/b*), and whether these associations are less strong for people who are more concerned about in-group identity continuity (*Hypothesis 4a/b*).

In testing these predictions, we included several control variables that have been found to be associated with tolerance and prejudice: level of education (e.g., Coenders & Scheepers, 2003), political orientation (e.g., Bansak et al., 2016), national identification (e.g., Gieling et al., 2014), religious affiliation (e.g., Van der Noll & Saroglou, 2015), age (e.g., O’Rourke & Sinnott, 2006) and gender (e.g., Van Doorn, 2014). When examining the relationships with acceptance of minority practices in Study 3, we additionally included prejudice as a control variable to assess the unique statistical associations of the two forms of tolerance, over and above prejudice.

## Study 1

The aim of Study 1 was to test whether the two forms of tolerance can be empirically distinguished and are independently related to prejudice. We tested these predictions in relation to tolerance of other people in general who ‘have a dissenting way of life’ (Study 1a) and subsequently referring to ‘the way of life of cultural minorities’ (Study 1b). This allowed us to assess the generality of the distinction between the more principled and pragmatic forms of tolerance and their relation with prejudice.

### Method

#### Data and Participants

In Study 1a, a sample of 1050 Dutch majority members participated with consent in an online survey. Potential respondents were selected from the *Kantar Public* consumer panel for fieldwork in the Netherlands (response rate 51%). From this online panel, a national sample of the ethnic Dutch population aged 18 years and older was compiled via a stratification procedure based on gender, age, education, household size and region. Four participants who identified as Muslim were excluded (*N* = 1046). Approximately half of the sample (51%) was female, and participants were between 18 and 85 years old (*M* = 47.00, *SD* = 15.67).

In Study 1b, a different sample of Dutch majority members was selected by *Kantar Public* from the *TNS NIPO* database to complete an online survey, consisting of eight version that were randomly presented to the participants (response rate 48%). Two versions of the survey contained the tolerance measures, which resulted in *N* = 218. Eight participants were excluded from the analyses because they indicated that their parents were not Dutch, resulting in an analytical sample of 210. Approximately half of the sample (51%) was female, and participants were between 18 and 85 years old (*M* = 51.47, *SD* = 16.71).

#### Measures

In Studies 1a and 1b, the *two forms of tolerance* were measured with three items each, using 7-point Likert-scales for answering (1 = *strongly disagree*, 7 = *strongly agree*). Some of the items were adapted from Klein and Zick (2013), who used items that showed relatively low reliabilities (α = 0.50 to α = 0.62). Therefore we developed additional items that focused more directly on tolerance by using a similar stem (see Table A1 in supplementary material). In Study 1a, the items were measured in relation to no specific group, and were preceded by the following introductory text: ‘Imagine that the dissenting way of life of certain people is rejected by the majority of the population. Below are reasons why these people may still live their life as they wish (within the confines of the law). To what extent do you agree or disagree with these reasons?’. In Study 1b, tolerance was measured in relation to cultural minorities in the Netherlands and the items were preceded by: ‘Below are several reasons for tolerating dissenting ways of life of cultural minorities. To what extent do you agree or disagree with each of these reasons?’. A sample item for *respect tolerance* is ‘…because they have the equal right to live their own life’ (α = 0.96 in Study 1a, and α = 0.91 in Study 1b), and a sample item for *coexistence tolerance* is ‘… in order to avoid social conflict’ (α = 0.93 in Study 1a, and α = 0.90 in Study 1b).

**Table 1:**
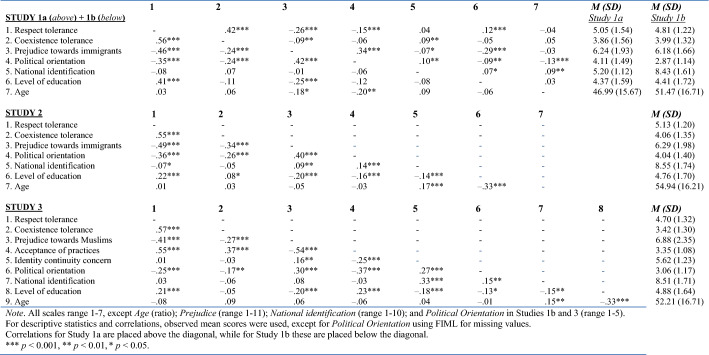
Means, standard deviations and correlations of continuous variables in Study 1a (*N* = 1046), Study 1b (*N* = 210), Study 2 (*N* = 824), Study 3 (*N* = 411)

*Prejudicial feelings* were measured with the well-known ‘feeling thermometer’ that ranged from 1 (0°, *very cold feelings*) to 11 (100°, *very warm feelings*), with 50° explicitly indicated as neutral feelings. Using feeling thermometers with wider ranges of responses than Likert-type scales generates a more reliable measure (Alwin, 1997), and these explicit measures tend to correlate with subtler methods of assessing prejudice (Dovidio et al., 2001). In Study 1a, respondents indicated their warm or cold feelings towards nine minority groups in the Netherlands: immigrants, refugees, Muslims, Poles, Rumanians, Turks, Moroccans, Antilleans and Surinamese in the Netherlands, and the combined items formed a reliable scale (α = 0.94). In Study 1b, the minority target groups were: Rumanian, Bulgarian, Polish, Turkish, Moroccan and Muslim, refugees and asylum seekers in the Netherlands (α = 0.93). All items were recoded so that a higher score indicated higher prejudice.

Besides *age* (continuous variable), *gender* (0 = men, 1 = women) and *religious affiliation* (0 = no affiliation, 1 = religious), we measured three other constructs that were used as control variables. *National identification* was assessed in Study 1a with two items (‘I identify with the Netherlands’ and ‘I feel connected to other Dutch people’; *r* = 0.57) on 7-point scales, and in Study 1b with a single item that has been shown to be a reliable and valid measure: ‘How strongly do you feel Dutch?’ (1 = *not at all,* 10 = *completely*) (Postmes et al., 2013). *Political orientation* was assessed with the well-known self-placement question (Jost, 2006) with a scale (7-point in Study 1a, and 5-point in Study 1b) ranging from a (strongly) left orientation via a center to a (strongly) right orientation, and was treated as a continuous variable in the analyses. Last, for assessing *level of education*, in both studies, participants indicated their highest educational achievement on a scale ranging from 1 (*no/only primary school*) to 7 (*master degree at (applied) university level*). The distinction between these levels of achieved education is comparable to the international ISCED measure that is used, for instance, in the European Social Survey. Similar to other research in the Netherlands (e.g., Van Tubergen & Van de Werfhorst, 2007), education was treated as a continuous variable in the analysis.

#### Analytic Strategy

We first used confirmatory factor analysis (CFA) in M*plus* version 7.3 (Muthén & Muthén, 1998–2012) to examine whether the items load onto the three latent constructs coexistence and respect tolerance, and prejudice. Subsequently, for examining the associations between the variables—without making any claims about a direction of influence—we specified a structural equation model in M*plus* in which prejudice was regressed on the two tolerance forms, and we controlled for the (manifest and mean-centered) control variables. Descriptive statistics and correlations were retrieved from SPSS 24.0.

There were no missing values on the key variables of interest, but in Study 1a there were missing values on the control variables political orientation (*N* = 144) and religious affiliation (*N* = 20). In Study 1b, there were only missing values for political orientation (*N* = 30). The missing values were dealt with in Mplus by using full information maximum likelihood (FIML; Graham, 2003).

### Results

#### Two Forms of Tolerance

In Study 1a, results of the CFA demonstrated that a three-factor model had a good fit to the data, χ^2^(86) = 644.40, *p* < 0.001; CFI = 0.96; TLI = 0.95; RMSEA = 0.08 [0.07–0.09]; SRMR = 0.03. The model included one modification, letting the errors of two prejudice items covary, and all factor loadings were above 0.68 (Kline, 2016).^4^ Subsequently, we tested a series of alternative models, which fit the data significantly worse than the proposed model (see Table A2 in supplementary material).

In Study 1b, results of the CFA showed that the proposed three-factor structure needed modifications for the prejudice items to reach an acceptable fit to the data. We maintained the two forms of tolerance as first-order factors, and used a second-order factor for prejudice, with three meaningful lower-order factors loading onto the second-order factor (Muthén & Muthén 1998–2012). The three lower-order factors clustered the minority groups that were similar in origin, religion or migration background: Muslims, Turkish and Moroccan immigrants formed one factor; Rumanian, Bulgarian and Polish immigrants formed another; and refugees and asylum seekers formed a third factor. The second-order factor allowed us to use a single latent score for prejudice towards all eight immigrant minority groups, while accounting for the covariance within the factor. This resulted in an acceptable fit: χ^2^(71) = 202.89, *p* < 0.001; CFI = 0.95; TLI = 0.93; RMSEA = 0.09 [0.08–0.11]; SRMR = 0.06. All standardized factor loadings were above 0.75 (Kline, 2016).^5^ For this model, items were permitted to load only on the constructs they were proposed to measure. Subsequently, we tested a series of alternative models, which fit the data significantly worse than the proposed three-factor structure (see Table A3 in supplementary material). Thus, in line with *H1a*, in both Study 1a and Study 1b, the two forms of tolerance were empirically distinct constructs that were also distinct from prejudice.

#### Descriptive findings

Descriptive statistics for the main variables of both samples are shown in Table 1. On average, participants in Studies 1a and 1b endorsed respect tolerance more strongly than coexistence tolerance, *t*(1045) = 23.173, *p* < 0.001 (Study 1a) and *t*(209) = 9.959, *p* < 0.001 (Study 1b). Further, in both samples, respect and coexistence tolerance were positively associated, and both forms of tolerance related negatively to prejudice.

#### Relations Between Forms of Tolerance and Prejudice

We regressed prejudice on the two forms of tolerance (see Table 2). Adding the control variables to the analyses did not change the results (see Table A4 and Table A5 in the supplementary material). For both samples, the findings demonstrated that stronger respect tolerance was associated with lower prejudice (in line with *H2*) and that coexistence tolerance was not independently associated with prejudice.

**Table 2:** Standardized regression coefficients from regression analyses with prejudice as dependent variable and forms of tolerance as independent latent variables, for all studies

Form of tolerance	Study 1a (*N* = 1046)	Study 1b (*N* = 210)	Study 2 (*N* = 824)	Study 3 (*N* = 411)
Respect	–0.28 (.03)***	–0.52 (.08)***	–0.47 (.04)***	–0.40 (.05)***
Coexistence	0.03 (.04)	0.04 (.09)	–0.09 (.04)*	–0.05 (.05)
*R*^*2*^	0.07***	0.25***	0.28***	0.18***

*Note*. *** *p* < 0.001, * *p* < 0.05

## Study 2

Extending the findings of Study 1, the aim of Study 2 was to examine whether people consistently distinguish between and similarly interpret the two forms of tolerance in relation to different immigrant target groups (*H1b*). Additionally, we explored whether tolerance is equally endorsed in relation to these target groups. We used an experimental design, manipulating the immigrant target group in relation to which the tolerance questions were asked. Specifically, we made a distinction between four broad immigrant categories that are commonly used in Dutch public and political debates (Cinalli & Giugni, 2013; Gijsberts & Lubbers, 2009): the distinction between Western and non-Western immigrants, and the distinction between Muslim and non-Muslim immigrants. Lastly, we explored whether the relations between tolerance and prejudice were similar across the four immigrant categories.

### Method

#### Data and Participants

This study used an online survey which was collected by research organization *Ipsos,* which used the *GfK* panel to approach a gross sample of 1640 panel members (response rate 52%). Eighteen respondents were removed by *GfK* to assure data quality. Eight participants were excluded from data analysis because they indicated that they were Muslim or that both of their parents were not Dutch (resulting in *N* = 824). Half of the sample (51%) was female, and participants were between 18 and 88 years old (*M* = 54.94, *SD* = 16.21).

#### Design and Measures

For the survey-embedded experiment, participants were randomly assigned to one of the four conditions that differed from each other in terms of the immigrant target group (Western, non-Western; Muslim, non-Muslim immigrants). In all four conditions, participants were presented with the same introductory text, tailored towards the specific category of immigrants that ‘can (within the confines of the law) live as they wish’. The same set of items for measuring respect tolerance (α = 0.91) and coexistence tolerance (α = 0.89) was used as in Studies 1a and 1b.

For *prejudice towards immigrants* we again used the same ‘feeling thermometer’ which assessed feelings towards four immigrant groups: refugees, and Turkish, Moroccan and Muslim immigrants in the Netherlands (α = 0.90).

We measured the same six control variables (level of education, national identification, political orientation, age, gender and religious affiliation) with exactly the same single item questions as in Study 1b. *Political orientation* was measured as in Study 1a, on a 7-point answer scale. Moreover, *level of education* had one different answer category than in Study 1b, creating a higher mean (see Table 1).

#### Analytic Strategy

Analyses for the full sample (i.e., collapsing the four immigrant categories) were conducted first to test the same hypotheses as in Study 1. We used a CFA (in M*plus*) to examine whether the two tolerance forms and prejudice were empirically distinct constructs. Subsequently, we specified a structural equation model, regressing prejudice on the tolerance forms.

Second, the data were analyzed across the four categories by testing multiple-group models. CFAs for all separate groups were conducted to examine whether the two forms of tolerance had the same meaning across the target groups. Subsequently, measurement invariance analyses were performed to test whether the measures (e.g., meaning of items, means and regression coefficients) could be compared across the four target groups. Furthermore, Wald-tests were used to compare the mean level of endorsement across groups. Lastly, we specified a structural equation model, regressing prejudice on the two forms of tolerance and comparing these relations across the four target groups.

There were no missing values on the main dependent and independent variables, but there were missing values for the three control variables national identification (*N* = 2), religious affiliation (*N* = 10) and political orientation (*N* = 92). Again, those were dealt with in M*plus* by using FIML.

### Results

#### Two Forms of Tolerance

CFA for the full sample replicated the results of Studies 1a and 1b by showing that the two forms of tolerance were empirically distinct (*H1a)*. The proposed three-factor structure (respect tolerance, coexistence tolerance, prejudice^6^) had a good fit to the data without modifications, χ^2^(32) = 104.42, *p* < 0.001; CFI = 0.988; TLI = 0.983; RMSEA = 0.052 [0.041–0.064]; SRMR = 0.028, and all standardized factor loadings were above 0.74. The three-factor model fit the data significantly better than various two- and a single-factor model (see Table A6 in supplementary material).

Again, on average participants agreed more strongly with respect tolerance compared to coexistence tolerance, *t*(823) = 25.00, *p* < 0.001 (see Table 1). Similar to studies 1a and 1b, respect and coexistence tolerance were positively associated, and again, both related negatively to prejudice.

#### Relations of Tolerance With Prejudice

Table 2 shows the results of a structural model which regressed prejudice on the two forms of tolerance. Both latent forms of tolerance had an independent significant relation with prejudice. Similar to studies 1a and 1b, stronger endorsement of respect tolerance was associated with lower prejudice (*H2*). Additionally, in Study 2 coexistence tolerance was also independently but weakly associated with lower prejudice, but this association was not significant (*p* = 0.074) after adding the control variables (see Table A7 in supplementary material).

#### Two Forms of Tolerance Across Four Immigrant Target Groups

To ensure that participants who were asked about the four different target groups interpreted the tolerance questions similarly, we tested for measurement invariance of the two-factor structure (respect and coexistence tolerance). Measurement invariance was tested by means of a multiple-group CFA, consecutively testing metric, scalar, and full uniqueness invariance (Van de Schoot et al., 2012). The model in which full invariance is assumed, had the lowest value for the Akaike (AIC) and Bayesian (BIC) index, indicating the best trade-off between model complexity and model fit (Van de Schoot et al., 2012). Moreover, CFI did not change more than 0.01 between the configural, metric, scalar and full invariance models, indicating that the threshold for full invariance was reached (Chen, 2007), which was confirmed by non-significant differences in χ^2^ between the models (see Table A8 in supplementary material). The other fit indices of this full invariance model also indicated a good fit. This means that the findings for the four immigrant target groups can be meaningfully compared on the latent tolerance constructs (e.g., mean scores, associations between the constructs across groups and with other variables across groups), which confirmed *H1b* (i.e., a similar distinctive meaning of each form of tolerance in relation to different immigrant target groups).

Comparing the mean tolerance scores between the four immigrant categories (see Table A9 in supplementary material) indicated that there were no significant differences in mean scores for respect tolerance (*Wald*(3) = 0.53, *p* = 0.913), and for coexistence tolerance (*Wald*(3) = 6.50, *p* = 0.090). Thus the two forms of tolerance were equally endorsed in relation to the four immigrant target groups.

#### Relations of the Tolerance Forms with Prejudice Across Target Groups

In order to test whether the tolerance-prejudice relationships were the same across the four target groups, we estimated a multiple-group comparison in our structural equation model (see Table A10 in supplementary material). Wald-tests showed that there were no differences across the four immigrant categories in the respect-prejudice relation (*Wald*(3) = 2.19, *p* = 0.534) and the coexistence-prejudice relation (*Wald*(3) = 1.83, *p* = 0.608).

## Study 3

Study 3 specifically focused on Muslims as the immigrant-origin group that is most strongly debated and negatively evaluated in Dutch society (Andriessen, 2016). The first aim was to examine whether the two forms of tolerance are associated with the acceptance of concrete Muslim minority practices, independently of the level of prejudice (*H3a and H3b*). Additionally, we examined whether these associations depend on the degree of concern about in-group identity continuity (*H4a and H4b).*

### Method

#### Data and Participants

A total of 815 respondents participated in the study after being drawn from a representative pool of ethnic Dutch. Data were collected with an online survey by research agency *GfK*, with a response rate of 54%. One case was excluded because the person self-identified as a Muslim. Since the questions about acceptance of concrete practices were asked to only half of the sample, this resulted in an analytical sample of *N* = 411. Similar to the previous studies, the sample consisted of 50% women and participants were between 18 and 92 years old (*M* = 52.21, *SD* = 16.71).

#### Measures

*Respect* (α = 0.90) and *coexistence* (α = 0.90) *tolerance* were measured with the same items as in Study 1b.

*Prejudice towards Muslims* was again measured with a feeling thermometer, but this time only towards Muslims in the Netherlands.

*Acceptance of Muslim minority practices* was measured with three items (7-point scales; 1 = *strongly disagree*, 7 = *strongly agree*) that involve practices which have triggered strong public debates and have been used in previous research in the Netherlands (Adelman & Verkuyten, 2020): ritual slaughter of animals by Muslims, Muslim public school teachers wearing a headscarf, and the building of new mosques. A higher score indicated greater acceptance (α = 0.73).

*Identity continuity concern* was assessed with three items (on the same 7-point scales) that were adapted from previous research (Smeekes & Verkuyten, 2015), and reflect a concern about the maintenance of Dutch cultural identity. We focus on identity continuity concern as a possible boundary condition for acceptance and the items were formulated to reflect this: ‘immigrants in the Netherlands can live as they wish as long as Dutch culture is preserved’, ‘…if Dutch traditions continue to exist’, and ‘…as long as Dutch identity is not affected’. These were combined into a mean score (α = 0.92), with a higher score indicating more concern for identity continuity.

The same measures as in the previous studies were used for the control variables *age* (continuous variable), *gender* (0 = men, 1 = women) and *religious affiliation* (0 = no affiliation, 1 = religious). For *national identification* and *political orientation* we again used the single item measures and scales as in Study 1b, and for *level of education,* the same question and answer scale was used as in Study 2.

Missing values (*N* = 109) for the control variable political orientation were dealt with by using FIML. For the moderation model, we included two (latent) interaction terms, and followed up with simple slope analysis (Aiken, West, & Reno, 1991) using M*plus*.

### Results

#### Two Forms of Tolerance

We first used CFA for testing *H1a* and whether the two forms of tolerance, continuity concern, and acceptance of concrete practices represented empirically distinct constructs. The four-factor structure had an acceptable fit to the data, χ^2^(47) = 163.29, *p* < 0.001; CFI = 0.965; TLI = 0.951; RMSEA = 0.078 [0.065–0.091]; SRMR = 0.048. Modification indices suggested allowing the errors between two of the coexistence items to covary, and all standardized factor loadings were above 0.60.^7^ Subsequently, we tested all possible alternative three-, two- and one-factor models and these fit the data significantly worse than the proposed four-factor structure (see Table A11 in supplementary material).

#### Descriptive Findings

Similar to Studies 1 and 2, participants more strongly endorsed respect tolerance than coexistence tolerance, *t*(410) = 21.248, *p* < 0.001. Furthermore, respect and coexistence tolerance were again positively associated, and related negatively to prejudice (see Table 1).

#### Relations of the Tolerance Forms with Prejudice

The results from the structural equation model confirmed that respect tolerance was again negatively related to prejudice (*H2*, see Table 2). Similar to Study 1, coexistence did not independently relate to prejudice towards Muslims. Including the control variables did not change these results (see Table A12 in supplementary material).

#### Relations of the Tolerance Forms with Acceptance

The findings from the structural equation model confirmed that respect tolerance was positively related to acceptance of Muslim practices (*β* = 0.45, SE = 0.06, *p* < 0.001), while controlling for prejudice towards Muslims (*H3a*). Coexistence tolerance did not independently relate to acceptance of concrete practices (*β* = 0.05, SE = 0.06, *p* = 0.444).^8^ Including the control variables did not change these results (see Table A13 in supplementary material).

#### Moderation of Identity Continuity Concern

The findings from the moderation model with respect and coexistence tolerance, continuity concern and their interactions,^9^ demonstrated that stronger concern about identity continuity was independently associated with lower acceptance (unstandardized *b* = –0.22, SE = 0.04, *p* < 0.001). More importantly, there was a significant interaction between respect and continuity concern, in line with *H4a* (*b* = –0.07, SE = 0.04, *p* = 0.042), but not between coexistence and continuity concern (*b* = 0.02, SE = 0.04, *p* = 0.694), controlling for prejudice towards Muslims.

As expected, simple slope analysis probing the respect by continuity interaction indicated that at low continuity concern (–1 *SD*), the relation between respect tolerance and acceptance of practices was stronger (*β* = 0.49, *p* < 0.001) than at high continuity concern (+ 1 *SD*: *β* = 0.31, *p* < 0.001).

## Discussion

Growing diversity and continuing immigration has led to an increased interest in social tolerance among policy makers and within the public and academic community. However, there can be different reasons for people to be tolerant of immigrant and minority groups, and we have focused on conceptualizing and measuring two key forms of tolerance that can be present in society at the same time: coexistence and respect tolerance (Forst, 2013, 2017). Both forms emphasize that cultural ‘others’ should be able to live the life that they want, but for different reasons.

Among four national majority samples in the Netherlands we clearly found that the two forms of tolerance are empirically distinct in relation to dissenting people in general and immigrant minorities in particular. Further, using an experimental design (Study 2), we demonstrated that each form had a similar meaning and similar levels of endorsement in relation to various immigrant categories that feature in public and political debates (Western, non-Western, Muslim and non-Muslim immigrants). This means that the measures can be used to examine and compare both forms of tolerance across different immigrant target groups, and that both forms are more general rather than group-specific conceptions of tolerance.

The distinction between the two forms of tolerance was further supported by their different relations with prejudice. As expected, across the four studies, we found a robust independent negative relation between respect tolerance and prejudice. Although coexistence tolerance was also negatively correlated with prejudice, this form of tolerance only had an independent and weak association with prejudice in Study 2. This pattern of findings demonstrates that tolerance is a phenomenon distinct from prejudice, which is in line with theoretical arguments in philosophy (Forst, 2013) and social psychology (Verkuyten et al., 2020), as well as other empirical research (Fairlamb & Cinnirella, 2020; Hjerm et al., 2019; Klein & Zick, 2013). Additionally, it indicates that the association between tolerance and prejudice differs for the two forms of tolerance. This means that the distinction between the two forms of tolerance might help to explain why some studies have found a relatively strong (negative) association between tolerance and prejudice (e.g., Helbling, 2014), while others have found no or only a weak association (Crawford, 2014; Van der Noll et al., 2010). Considering the different reasons for tolerance allows for a more detailed understanding of the difference between tolerance and prejudice and the extent to which these co-occur.

Higher respect tolerance was found to be associated with lower prejudice and was endorsed relatively strong in the four national samples. Thus, majority members tended to agree with the principle that immigrants are autonomous citizens with equal rights who have the freedom to live the life that they want. Furthermore, stronger respect tolerance was not only a general abstract belief, but was also associated with the acceptance of concrete Muslim minority practices (Study 3). Thus, respecting immigrants as fellow citizens translated into higher acceptance of concrete practices, which is in line with research in other Western countries (Hjerm et al., 2019; Simon et al., 2018).

The importance of respect tolerance for the acceptance of minority practices is further demonstrated by the fact that the association was also positive for individuals who were concerned about the continuity of their in-group culture and identity. Yet, perceived concern about identity continuity did weaken the association between respect tolerance and acceptance. Acceptance is not without its boundaries and it appears to be more difficult to accept Muslim minority practices when the continuity of the national identity is considered to be at stake (Verkuyten et al., 2019). Although identity continuity has been examined in relation to negative outgroup attitudes (Jetten & Wohl, 2012; Smeekes & Verkuyten, 2013), to our knowledge, this is one of the first studies that demonstrated that identity continuity can be a boundary condition to accepting minority practices.

Coexistence tolerance was negatively correlated with prejudice (all studies) and positively associated with the acceptance of Muslim practices (Study 3). Higher coexistence tolerance was also quite strongly associated with higher respect tolerance (see also Klein & Zick, 2013). This indicates that people who consider it important to tolerate minorities for reasons of peaceful cohabitation and societal harmony, also tend to respect them as equal citizens. However, the latter appears to be more important than the former, because coexistence tolerance was only in Study 2 independently related to prejudice, and was not independently related to the acceptance of Muslim minority practices. One possible explanation is that the more instrumental, pragmatic nature of coexistence tolerance makes it less morally imperative than respect tolerance, and therefore more contextual, resulting in a less strong relation with prejudicial feelings and the acceptance of concrete minority practices.

### Limitations and Future Directions

Despite our novel contribution to the study of tolerance, there are several limitations to our research that provide directions for future studies. First, the studies were conducted among Dutch majority members and it remains to be seen whether the results can be generalized across countries (see Hjerm et al., 2019; Klein & Zick, 2013). Each country has its specific history of immigration and ways for dealing with diversity, and future research in other contexts should address to what extent context characteristics are relevant for the meaning and importance of the two forms of social tolerance.

Second, we have not examined our measures of tolerance in relation to other ways of measuring social tolerance (e.g. Hjerm et al., 2019), including tolerance of practices and beliefs that one explicitly dislikes or disapproves of (e.g., Sleijpen et al., 2020). This comparison was beyond the scope of our research and could be addressed in future studies. Furthermore and similar to other studies (Adelman & Verkuyten, 2020; Sniderman & Hagendoorn, 2007), we investigated the acceptance of three Muslim minority practices, but the relation between tolerance and acceptance of minority practices might vary according to the specific type of practice and target minority group (e.g., sexual minorities), which could be examined in future research.

Third, it should be noted that in Study 2 the experimental manipulation about the different target groups involved the use of four broad category labels. Although the distinctions between these broad categories are widely discussed in public and policy debates, all four may have triggered similar stereotypical beliefs about newcomers. Thus, it remains to be seen whether the findings are similar if more specific group labels would be used (e.g. Polish rather than western immigrants, and Somali instead of non-western immigrants). Further, for the experimental manipulation, participants read a short introduction in an online survey, and it is possible that more extensive (personal narratives) and vivid (visual, auditive) manipulations would show target-group differences in the two forms of tolerance. However, simply mentioning these broad category labels is common in the media and in public and political debates and therefore close to empirical reality.

### Conclusion

To conclude, we have tried to advance the study of social tolerance by clarifying two main forms and demonstrating that these can be assessed in a reliable and valid way. We found that in relation to various groups, people make a consistent distinction between respect and coexistence tolerance. Furthermore, these two are relatively independent of group-based prejudice, and have different relations with the acceptance of concrete minority practices. Overall, respect tolerance was found to relate to more positive attitudes towards minority groups and their practices, while coexistence tolerance had no clear independent beneficial outcomes for minority groups. Although tolerating to avoid conflicts may in some situations be the best possible option, it remains a pragmatic and not a principled solution. Stimulating respect tolerance—for instance by emphasizing equal rights for all citizens in educational programs—might be particularly helpful for improving intergroup relations in culturally diverse societies. Furthermore, it might form a stepping stone towards full recognition, or as Goethe (in Forst, 2017, p.3) famously said: ‘Tolerance should be a temporary attitude only: it must lead to recognition’.

## Supplementary Information

Below is the link to the electronic supplementary material.

Supplementary file1 (PDF 280 kb)

## Data Availability

The anonymized research data will be available to access (via Data Archiving and Networked Services) after the ERC research project has ended. Code availability: not applicable.

## Footnotes

1 We use the term social tolerance as opposed to political tolerance which centers on extending basic political rights to all members of society. Social tolerance refers to group members tolerating social and cultural practices which reproduce social identities (Verkuyten et al., 2019).

2 Forst (2013) also distinguished *esteem* and *permission tolerance* which we did not examine here*. Esteem tolerance* with its emphasis on valuing diversity is very similar to full recognition (Klein & Zick, 2013), and *permission tolerance* refers to the hierarchical intergroup context of tolerance rather than an underlying reason for being tolerant.

3 With the term “Muslim practices”, we refer to practices linked to Muslims in Western Europe and frequently debated in those societies, such as teachers wearing a headscarf in public schools. This is not to say that the practices are defining of, or undebated within Muslim communities.

4 A measurement model with only the tolerance items (two-factor structure) was also examined, and had a good fit to the data, χ^2^(8) = 15.07, *p* = 0.058; CFI = 1.00; TLI = 1.00; RMSEA = 0.03 [0.00–0.05]; SRMR = 0.01. The model included no modifications and all standardized factor loadings were above 0.90.

5 A two-factor measurement model with only the tolerance items had a good fit to the data, χ^2^(7) = 9.36, *p* = 0.223; CFI = 1.00; TLI = 1.00; RMSEA = 0.04 [0.00–0.10]; SRMR = 0.02. The model included one modification (letting the errors of two coexistence tolerance items covary), and all factor loadings were above .73.

6 A measurement with only the tolerance items had a good fit to the data, χ^2^(8) = 9.32, *p* = 0.316; CFI = 1.00; TLI = 1,00; RMSEA = 0.01 [0.00–0.05]; SRMR = 0.11. The model included no modifications and all factor loadings were above .83.

7 A measurement model with only the tolerance items had an acceptable fit to the data, χ^2^(7) = 38.96, *p* < 0.001; CFI = 0.98; TLI = 0.96; RMSEA = 0.11 [0.08–0.14]; SRMR = 0.03. The model included one modification (letting the errors of two coexistence tolerance items covary), and all factor loadings were above .74.

8 When analyzing a separate model for coexistence tolerance (controlling for prejudice), coexistence tolerance did show a significant positive relation to acceptance,* β* = 0.29, SE = 0.05, *p* < 0.001.

9 When analyzing two separate models this led to similar results: a significant interaction between respect and continuity, and no significant interaction between coexistence and continuity (controlling for prejudice).
