# Supplementary material for: The Different Faces of Social Tolerance: Conceptualizing and Measuring Respect and Coexistence Tolerance
Source: Soc Indic Res. 2021 Jun 16;158(3):1105–25. doi: 10.1007/s11205-021-02724-5 (PMC8572818; doi:10.1007/s11205-021-02724-5)
Supplement: Supplementary file 1 — Supplementary file1 (PDF 280 kb) [file 11205_2021_2724_MOESM1_ESM.pdf]

SUPPLEMENTARY MATERIAL to 'The different faces of social tolerance'

Table A1. Items used to construct the latent variables for the two forms of tolerance, Studies 1a, 1b and 3 (for Study 2, adapted versions of the below items were used in an experimental manipulation).

Introduction:

'Cultural diversity can be seen as enriching the Netherlands, but might also mean that ways of life collide. Below are several reasons for either accepting or rejecting immigrants' ways of life. To what extent do you agree or disagree with each of these reasons?'

Respect tolerance

1. Immigrants in the Netherlands can live as they wish because they have the right to live their own life
2. Immigrants in the Netherlands can live as they wish because they should be able to enact their own identity
3. Immigrants in the Netherlands can live as they wish because they should be able to practice their own religion in freedom

Coexistence tolerance

4. Immigrants in the Netherlands can live as they wish in order to ensure that there are less social tensions
5. Immigrants in the Netherlands can live as they wish in order to avoid social conflict
6. Immigrants in the Netherlands can live as they wish in order to maintain peace in society

---

Table A2. Model fit indices of competing measurement models, Study 1a (*N* = 1046).

**Model**	**χ^2 (df)**	**$\Delta\chi^2$**	**CFI**	**TLI**	**RMSEA [CI]**	**SRMR**
3 factors	644.40 (86)***	-	.96	.95	.08 [0.07-0.08]	.03
2 factors **A**[1]	3750.00 (88)***	3105.60***	.74	.69	.20 [0.19-0.21]	.12
2 factors **B**[2]	4197.14 (88)***	3552.74***	.70	.65	.21 [0.21-0.22]	.15
2 factors **C**[3]	3314.64 (88)***	2670.2***	.77	.72	.19 [0.18-0.19]	.15
1 factor[4]	6668.29 (89)***	6023.89***	.53	.44	.27 [0.26-0.27]	.19

Note. CFI: comparative fit index; TLI: Tucker-Lewis fit index; RMSEA: root-mean-square error of approximation; CI: 90% confidence interval; SRMR: standardized root mean squared residual.
*** $p < .001$.

[1] In model 2a, the respect scale and coexistence tolerance scales were forced to load onto one factor, while prejudice was a separate factor. This was compared to the three-factor model.
[2] In model 2b, the respect and prejudice scales were forced to load onto one factor, while coexistence tolerance was a separate factor. This was compared to the three-factor model.
[3] In model 2c, the coexistence and prejudice scales were forced to load onto one factor, while respect tolerance was a separate factor. This was compared to the three-factor model.
[4] In model 1, the prejudice, coexistence and respect tolerance scales were combined and forced to load onto one factor, which was compared to the three-factor model.

Table A3. Model fit indices of competing measurement models, Study 1b (*N* = 210).

**Model**	**χ^2 (df)**	**$\Delta\chi^2$**	**CFI**	**TLI**	**RMSEA [CI]**	**SRMR**
3 factors[5]	202.89 (71)***	-	.95	.93	.09 [0.08-0.11]	.06
2 factors **A**[6]	473.97 (73)***	271.08***	.84	.80	.16 [0.15-0.18]	.08
2 factors **B**[7]	888.79 (76)***	685.90***	.68	.62	.23 [0.21-0.24]	.15
2 factors **C**[8]	864.71 (76)***	661.82***	.69	.63	.22 [0.21-0.24]	.16
1 factor	1270.40 (77)***	1067.51***	.53	.45	.27 [0.26-0.29]	.17

Note. CFI: comparative fit index; TLI: Tucker-Lewis fit index; RMSEA: root-mean-square error of approximation; CI: 90% confidence interval; SRMR: standardized root mean squared residual.
*** $p < .001$.

[5] As mentioned in the article, the three-factor model uses a second-order factor for prejudice.
[6] In model 2a, the respect scale and coexistence scale were forced to load onto one factor, while prejudice was maintained as a separate factor. This is compared to the three-factor model.
[7] In model 2b, the respect and prejudice scales were forced to load onto one factor, while coexistence tolerance was maintained as a separate factor. This is compared to the three-factor model.
[8] In model 2c, the prejudice and coexistence scales were forced to load onto one factor, while respect tolerance was maintained as another factor. This was compared to the three-factor model.

Table A4. Standardized regression coefficients from regression analyses with prejudice as dependent latent variable and forms of tolerance as independent latent variables, including control variables, Study 1a (N = 1046).

	**Model 1: Model excluding control variables**	**Model 2: Model including control variables**
	Prejudice	Prejudice
Respect tolerance	–.28 (.03)***	–.19 (.03)***
Coexistence tolerance	.03 (.04)	.00 (.03)
**Control variables**		
Age		.01 (.03)
Religious affiliation (ref: *no*)		–.03 (.03)
Gender (ref: *male*)		–.06 (.03)*
Level of education		–.24 (.03)***
Political orientation		.31 (.03)***
National identification		–.06 (.03)*
R^2	.07***	.23***

Note. *** p < .001, * p < .05.

Table A5. Standardized regression coefficients from regression analyses with prejudice towards immigrants as dependent latent variable and forms of tolerance as independent latent variables, including control variables, Study 1b (N = 210).

	**Model 1: Model excluding control variables**	**Model 2: Model including control variables**
	Prejudice	Prejudice
Respect tolerance	–.52 (.08)***	–.40 (.09)***
Coexistence tolerance	.04 (.09)	.07 (.09)
**Control variables**		
Age		–.11 (.07)
Religious affiliation (ref: *no*)		–.07 (.06)
Gender (ref: *male*)		–.06 (.06)
Level of education		–.07 (.07)
Political orientation		.33 (.07)***
National identification		–.02 (.06)
R^2	.25***	.35***

Note. *** $p < .001$.
As the sample size was relatively small for structural equation modeling, additional analyses with manifest (rather than latent) variables were performed, which did not change the results.

Table A6. Model fit indices of competing measurement models, Study 2 (N = 824).

**Model**	**χ^2 (df)**	**$\Delta\chi^2$**	**CFI**	**TLI**	**RMSEA [CI]**	**SRMR**
3 factor	104.42 (32)***	-	.99	.98	.05 [0.04-0.06]	.03
2 factors **A**[9]	1034.53 (34)***	930.11***	.83	.78	.19 [0.18-0.20]	.09
2 factors **B**[10]	1620.99 (34)***	1516.57***	.73	.64	.24 [0.23-0.25]	.12
2 factors **C**[11]	1548.03 (34)***	1443.61***	.74	.66	.23 [0.22-0.24]	.16
1 factor	2536.32 (35)***	2431.90***	.58	.46	.30 [0.29-0.30]	.15

Note. CFI: comparative fit index; TLI: Tucker-Lewis fit index; RMSEA: root-mean-square error of approximation; CI: 90% confidence interval; SRMR: standardized root mean squared residual.
*** p < .001.

[9] In model 2A, the respect and coexistence constructs were combined to load onto one factor, and prejudice formed a separate factor. This was compared to the three-factor structure.
[10] In model 2B, respect and prejudice were combined to load onto one factor, and coexistence formed a separate factor. This was compared to the three-factor model.
[11] In model 2C, coexistence and prejudice were combined to load onto one factor, and respect formed a separate factor. This was compared to the three-factor model.

Table A7. Standardized regression coefficients from regression analyses with prejudice towards immigrants as dependent latent variable and forms of tolerance as independent latent variables, including control variables, Study 2 (N = 824).

	**Model 1: Model excluding control variables**	**Model 2: Model including control variables**
	Prejudice	Prejudice
Respect tolerance	–.47 (.04)***	–.37 (.04)***
Coexistence tolerance	–.09 (.04)*	–.08 (.04)†
**Control variables**		
Age		–.07 (.03)*
Religious affiliation (ref: *no*)		–.06 (.03)*
Gender (ref: *male*)		.01 (.03)
Level of education		–.10 (.03)**
Political orientation		.24 (.04)***
National identification		.04 (.03)
R^2	.28***	.33***

Note. *** p < .001, ** p < .01, * p < .05, † p < .10.

Table A8. Measurement invariance comparing the tolerance items across the four experimental groups, Study 2 (N = 824).

	χ^2	df	CFI	TLI	RMSEA	BIC	AIC
Configural invariance[12]	61.95**	32	0.991	0.984	0.067	14299.97	13941.70
Metric invariance	67.61*	44[1]	0.993	0.991	0.051	14225.07	13923.36
Scalar invariance	83.89**	56[2]	0.992	0.991	0.049	14160.77	13915.64
Full invariance	109.13**	74[3]	0.990	0.992	0.048	14065.16	13904.88

Note. CFI = Comparative Fit Index; TLI = Tucker-Lewis Index; RMSEA = Root Mean Square Error of Approximation; BIC = Bayesian Information Criterion; AIC = Akaike Information Criterion.

** $p < .01$, * $p < .05$.

[1] $\Delta\chi^2$ (12) = 5.66, p = .932 indicates that there was no significant difference between the configural and metric measurement invariance models.

[2] $\Delta\chi^2$ (12) = 16.28, p = .179 indicates that there was no significant difference between the metric and scalar measurement invariance models.

[3] $\Delta\chi^2$ (18) = 25.24, p = .118 indicates that there was no significant difference between the scalar and full measurement invariance models.

[12] Four CFAs were also conducted separately for each experimental group, and the model had a good fit in all separate groups:
For the group that was asked about *Western* immigrants (n = 202), the proposed two-factor structure had a good fit to the data, $\chi^2(8)$ = 15.939, $p < .05$; CFI = 0.991; TLI = 0.983; RMSEA = 0.070 [0.012-0.120]; SRMR = 0.035 (AIC = 3418.66, BIC = 3481.51).
For the group that was asked about *non-western* immigrants (n = 207), the proposed two-factor structure also had a good fit to the data, $\chi^2(8)$ = 7.667, p = .466; CFI = 1.00; TLI = 1.00; RMSEA = 0.000 [0.000-0.079]; SRMR = 0.019 (AIC =3439.88, BIC = 3503.21).
For the group that was asked about *Muslim* immigrants (n = 208), the proposed two-factor structure again had an acceptable to good fit to the data, $\chi^2(8)$ = 23.46, $p < .01$; CFI = 0.984; TLI = 0.970; RMSEA = 0.096 [0.053-0.143]; SRMR = 0.033 (AIC = 3539.88, BIC = 3603.29).
Lastly, for the group that was asked about *non-Muslim* immigrants (n = 207), the proposed two-factor structure again had a good fit to the data, $\chi^2(8)$ = 14.88, p = .062; CFI = 0.991; TLI = 0.983; RMSEA = 0.064 [0.000-0.115]; SRMR = 0.029 (AIC =3543.28, BIC = 3606.60).

Table A9. Mean scores and standard deviations for the two forms of tolerance, per type of immigrant group, Study 2 (N = 824).

	**Western** (n = 202)	**non-Western** (n = 207)	**Muslim** (n = 208)	**non-Muslim** (n = 207)
	M (SD)	*M (SD)*	*M (SD)*	*M (SD)*
Respect tolerance	5.12 (1.19)	5.14 (1.16)	5.09 (1.32)	5.16 (1.15)
Coexistence tolerance	4.13 (1.38)	3.86 (1.36)	4.08 (1.37)	4.18 (1.30)

Note. Observed mean scores and standard deviations are reported.

Table A10. Standardized regression coefficients from multiple-group regression analyses (*constrained model*) with prejudice towards immigrants as dependent latent variable and forms of tolerance as independent latent variables, Study 2 (N = 824).

	**Western** (*n* = 202)	**non-Western** (*n* = 207)	**Muslims** (*n* = 208)	**non-Muslims** (*n* = 207)
Respect tolerance	–.46 (.05)***	–.44 (.05)***	–.50 (.05)***	–.43 (.05)***
Coexistence tolerance	–.11 (.05)*	–.11 (.05)*	–.10 (.04)*	–.10 (.04)*
R^2	.28***	.26***	.34***	.25***

Note. *** $p < .001$, * $p < .05$.
An unconstrained model (i.e., tolerance-prejudice relations vary across the four immigrant categories) did not fit better than a constrained model (i.e., tolerance-prejudice relations are forced to be equal across groups), χ^2 (6) = 6.55, p = .364. This indicates that there are no significant differences between the four groups on the tolerance-prejudice relations, and thus the constrained model is reported here.

Table A11. Model fit indices of competing measurement models, Study 3 (N = 411).

Model	χ^2 (df)	$\Delta\chi^2$	CFI	TLI	RMSEA [CI]	SRMR
4 factors	163.29 (47)***	-	.97	.95	.08 [0.07-0.09]	.05
3 factors **A**[13]	355.31 (50)***	192.02***	.91	.88	.12 [0.11-0.13]	.07
3 factors **B**[14]	335.80 (50)***	172.51***	.91	.89	.12 [0.11-0.13]	.08
3 factors **C**[15]	420.38 (50)***	257.09***	.89	.85	.13 [0.12-0.15]	.08
3 factors **D**[16]	1113.73 (50)***	950.42***	.68	.58	.23 [0.22-0.24]	.16
2 factors **A**[17]	1243.63 (52)***	1080.34***	.64	.55	.24 [0.23-0.25]	.16
2 factors **B**[18]	1340.96 (52)***	1177.67***	.61	.51	.25 [0.23-0.26]	.17
2 factors **C**[19]	1305.25 (52)***	1141.96***	.62	.52	.24 [0.23-0.25]	.17
2 factors **D**[20]	525.97 (52)***	362.68***	.86	.82	.15 [0.14-0.16]	.10
2 factors **E**[21]	772.63 (52) ***	609.34***	.78	.73	.18 [0.17-0.20]	.19
1 factor	1431.98 (53)***	1268.69***	.59	.49	.25 [0.24-0.26]	.17

Note. CFI: comparative fit index; TLI: Tucker-Lewis fit index; RMSEA: root-mean-square error of approximation; CI: 90% confidence interval; SRMR: standardized root mean squared residual.
*** p < .001.

[13] In model 3a, the respect and coexistence scale were forced to load onto one factor. The alternative models were compared to the four-factor model.
[14] In model 3b, the respect and acceptance scales were forced to load onto one factor.
[15] In model 3c, the acceptance and coexistence scales were forced to load onto one factor.
[16] In model 3d, the coexistence and continuity scales were forced to load onto one factor.
[17] In model 2a, the coexistence and continuity scales were combined and forced to load onto one factor, and respect and acceptance were combined to load onto a second factor.
[18] In model 2b, the coexistence and acceptance scales were combined and forced to load onto one factor, and respect and continuity were combined to load onto a second factor.
[19] In model 2c, the coexistence, respect and continuity scales were forced to load onto one factor, and acceptance was forced to load onto a second factor.
[20] In model 2d, coexistence, respect and acceptance were forced to load onto one factor, and continuity was forced to load onto a second factor.
[21] In model 2e, respect and coexistence tolerance were forced to load onto one factor, and the other factor was acceptance combined with continuity

Table A12. Standardized regression coefficients from regression analyses with prejudice towards Muslims as manifest dependent variable and forms of tolerance as independent latent variables, including control variables, Study 3 (*N* = 411).

	**Model 1: Model excluding control variables**	**Model 2: Model including control variables**
	Prejudice	Prejudice
Respect tolerance	–.35 (.06)***	–.26 (.07)***
Coexistence tolerance	–.10 (.07)	–.13 (.07)†
**Control variables**		
Age		–.01 (.05)
Religious affiliation (ref: *no*)		–.13 (.05)**
Gender (ref: *male*)		–.09 (.05)
Level of education		–.15 (.05)**
Political orientation		.20 (.05)***
National identification		.03 (.05)
R^2	.18***	.25***

Note. *** p < .001, ** p < .01, † p < .10.

Table A13. Standardized regression coefficients from regression analyses with acceptance as dependent latent variable and forms of tolerance as independent latent variables, including manifest control variables, Study 3 (*N* = 411).

	**Model 1: Model excluding control variables**	**Model 2: Model including control variables**
	Acceptance	Acceptance
Respect tolerance	.59 (.06)***	.39 (.07)***
Coexistence tolerance	.08 (.07)	.08 (.07)
**Control variables**		
Age		.02 (.05)
Religious affiliation (ref: *no*)		.02 (.04)
Gender (ref: *male*)		.04 (.05)
Level of education		.14 (.05)**
Political orientation		–.25 (.05)***
National identification		.02 (.04)
Prejudice Muslims		–.35 (.05)***
R^2	.42***	.59***

Note. *** $p < .001$, ** $p < .01$.
